# Harvesting Time Modulates Starch Structure, Physicochemical Properties and In Vitro Digestibility of Wuyoudao4 Japonica Rice

**DOI:** 10.3390/foods15081307

**Published:** 2026-04-09

**Authors:** Yiming Wei, Fanchong Ren, Jiayu Cui, Ming Gao, Fei Jia, Xiaowei Zhang, Yapeng Fang

**Affiliations:** 1 School of Health Science and Engineering, University of Shanghai for Science and Technology, Shanghai 200093, China; 232332345@st.usst.edu.cn (Y.W.); ren223392711@163.com (F.R.); 233442704@st.usst.edu.cn (J.C.); ypfang@sjtu.edu.cn (Y.F.); 2 Wilmar (Shanghai) Biotechnology Research and Development Center Co., Ltd., Pudong New District, Shanghai 200137, China; gaoming5@cn.wilmar-intl.com; 3 College of Food Science and Engineering, Ocean University of China, Qingdao 266003, China; feijia@ouc.edu.cn; 4 Department of Food Science and Engineering, School of Agricultural and Biology, Shanghai Jiao Tong University, Shanghai 200240, China

**Keywords:** harvest time, starch structure, physicochemical properties, in vitro digestibility

## Abstract

Harvesting time is an important but insufficiently characterized factor affecting rice starch structure and digestibility. Wuyoudao4 (WYD4) rice was harvested weekly from 45 to 87 days after heading to investigate changes in starch structure, physicochemical properties, and in vitro digestion. With delayed harvesting, starch crystallinity of cooked rice decreased from 6.27% to 4.97%, while total amylase and α-amylase activities increased significantly. Rice flour particle size (D10 and D90) was reduced after mid-harvest, indicating improved enzyme accessibility. Consequently, starch digestibility at 120 min increased from 80.53% (week 1) to 95.60% (week 7). Kinetic analysis confirmed a single-stage digestion mechanism, with enzyme binding as the rate-limiting step and higher digestion extent at later harvest time. These results demonstrate that harvest time modulates rice starch digestibility through coordinated changes in crystallinity, enzymatic activity, and particle accessibility, providing new mechanistic insight into harvest-dependent starch digestion behavior.

## 1. Introduction

Rice (*Oryza sativa* L.) is one of the most important cereal crops worldwide and serves as the staple food for more than half of the global population [1]. In China, rice contributes to the daily caloric intake of approximately 65% of the population [2]. The rice growth cycle generally consists of four major stages, including vegetative growth, heading, grain filling, and physiological maturity [3,4]. After heading, rice grains enter the grain-filling stage, during which starch and protein continuously accumulate in the endosperm [5,6,7]. This process usually lasts 30–50 days, depending on cultivar and environmental conditions [4].

For japonica rice cultivated in cold regions such as Northeast China, heading typically occurs from late July to early August, followed by a grain-filling and maturation period extending into September [8]. Rice grains gradually reach physiological maturity at approximately 40–45 days after heading (DAH), when dry matter accumulation stabilizes and the moisture content declines to a level suitable for harvesting [7]. Therefore, 40–50 DAH is generally considered the earliest time point at which rice becomes harvestable [7]. From this point onward, farmers may choose different harvest times depending on climatic conditions, labor availability, and expected grain quality.

In Northeast China, rice production is dominated by early-maturing, single-season japonica cultivars that are harvested only once per year. Consequently, the choice of harvest time represents a critical agronomic decision. Premature harvesting may result in incomplete grain filling, reduced thousand-grain weight, and inferior milling quality, whereas delayed harvesting can increase grain cracking, lodging risk, and post-harvest losses [9]. Thus, determining an optimal harvest window is essential for achieving both high yield and desirable grain quality. Previous studies have primarily focused on the effects of harvest time on yield-related traits and milling performance, including grain dry matter accumulation, head rice yield, and thousand-grain weight [10]. These studies generally report that rice yield increases initially and then decreases with delayed harvesting, allowing an optimal harvest time to be identified [11]. However, most of these investigations rely on macroscopic agronomic indicators, while the underlying quality formation mechanisms remain poorly understood. In contrast, systematic information regarding how harvest time influences the physicochemical and eating quality of rice is still limited. Key parameters such as starch molecular structure, crystallinity, enzymatic activity, pasting behavior, texture characteristics, and starch digestibility have rarely been integrated into harvest-time studies. These properties are directly related to rice cooking performance and consumer acceptability [6,12]. The lack of mechanistic understanding restricts the development of scientifically guided harvest strategies.

Here, we hypothesize that harvest time modulates rice eating quality by altering starch molecular structure and endogenous amylase activity, thereby regulating starch gelatinization behavior, texture properties, and digestion characteristics. To test this hypothesis, Wuchang wuyoudao4 (hereinafter abbreviated as WYD4), a representative high-quality aromatic japonica rice cultivar from Heilongjiang Province, was harvested at seven time points from 45 to 87 days after heading, covering the entire practical harvesting window. Rice collected at 45 DAH (week 1) represented the earliest harvestable stage, while subsequent samples were obtained at weekly intervals. A comprehensive analysis was conducted, including particle size distribution, starch chain length distribution, crystallinity, amylase activity, pasting properties, texture profile analysis, and in vitro starch digestion kinetics. This study aims to elucidate the physicochemical mechanisms underlying harvest-time-dependent quality formation in aromatic japonica rice. The findings are expected to provide a scientific basis for optimizing harvest time, improving eating quality, and reducing economic losses in rice production systems.

## 2. Material and Methods

### 2.1. Materials

WYD4 rice samples grown in Wuchang City, Heilongjiang Province, China, were harvested from 45 to 87 days after heading. Rice harvested at 45 days after heading was defined as week 1, and samples collected at weekly intervals up to week 7 were included. All rice grains were provided by Wilmar (Shanghai) Biotechnology Research & Development Center Co., Ltd. (Shanghai, China). Paddy rice samples were dehulled using standard mechanical hulling to remove the husk, followed by milling to obtain polished rice. No additional surface treatment was applied prior to milling. Isoamylase (200 U/mL), amyloglucosidase (3300 U/mL) and total starch (K-TSTA-100A) kits were obtained from Megazyme International, Ltd. (Bray, Co. Wicklow, Ireland). Porcine pancreatin (P1750-25G) was obtained from Sigma-Aldrich Chemical Co., Ltd. (St. Louis, MO, USA). Sodium hydroxide, 3,5-dinitrosalicylic acid (DNS) reagent, soluble starch, and all other analytical-grade chemicals used in the experiment were purchased from Shanghai Vita Chemical Reagent Co., Ltd. (Shanghai, China).

### 2.2. Sample Preparation

Rice samples were ground using a high-speed cyclone mill for five cycles of 20 s each and passed through a 100-mesh sieve. The resulting rice flour was used for determination of protein content, starch chain length distribution (CLDs), particle size distribution, amylase activity, and pasting properties and was stored at 4 °C until analysis.

For analyses requiring cooked rice, 100 g of milled rice was cooked in an electric rice cooker with a rice-to-water ratio of 1:1.3 (*w*/*v*) for 25 min, followed by standing for 20 min. The cooked rice was used directly for textural profile analysis (TPA). Subsequently, a portion of the cooked rice was freeze-dried and stored at 4 °C prior to X-ray diffraction (XRD) and in vitro starch digestibility analyses.

### 2.3. Determination of Protein Content

Protein content was determined in triplicate using the Kjeldahl method [13]. Approximately 0.6 g of rice flour was placed into a digestion tube with 6 g of catalyst (CuSO_4_·5H_2_O:K_2_SO_4_ = 1:15, *w*/*w*) and 12 mL of concentrated sulfuric acid. The mixture was digested at 450 °C for 2 h. After cooling, nitrogen content was determined using a Kjeltec 8400 Kjeldahl nitrogen analyzer (FOSS, Hillerød, Denmark). Protein content was calculated using a nitrogen-to-protein conversion factor of 6.25.

### 2.4. Starch CLDs of WYD4 Harvested at Different Times

Starch CLDs of rice samples harvested at different times were determined according to the previously reported method [14]. Briefly, 10 mg of rice flour was mixed with 0.5 mL of 0.45% (*w*/*v*) sodium bisulfite solution, vortexed, and stored at 4 °C for 12 h. The mixture was centrifuged at 4000 rpm for 10 min, and the supernatant was discarded. Subsequently, 0.5 mL of protein digestion solution (0.36 mg protease in Tricine buffer) was added, followed by incubation at 37 °C and 800 rpm for 12 h to remove proteins. After centrifugation, 1.5 mL of DMSO/LiBr solution was added, and the mixture was incubated at 80 °C and 800 rpm for 24 h to dissolve starch. The solution was centrifuged at 9000 rpm for 10 min, and the supernatant was precipitated with 10 mL ethanol, centrifuged, and air-dried. The washing step was repeated once. The dried precipitate was dispersed in 0.9 mL boiled ultrapure water and heated for 1 h. After cooling, 100 μL of sodium acetate buffer (pH 3.5), 2.5 μL isoamylase, and 1 μL ProClin were added, followed by incubation at 37 °C for 3 h. The reaction was terminated by adding 0.1 mL of 0.1 mol/L NaOH and heating at 80 °C for 1 h, after which samples were freeze-dried. The dried starch was redissolved in DMSO/LiBr to a concentration of approximately 4 mg/mL and analyzed using a Shimadzu LC-20AD size-exclusion chromatography (SEC) system equipped with a RID-10A refractive index detector (Shimadzu, Kyoto, Japan). Separation was performed using GRAM columns (pre-column, 1000 Å, and 100 Å) at a flow rate of 0.6 mL/min.

### 2.5. Determination of Particle Size

The particle size distribution of rice flour was determined using a Microtrac S3500 laser diffraction particle size analyzer (Microtrac Inc., Montgomeryville, PA, USA), following a previously reported method [15]. Samples were dispersed in deionized water under magnetic stirring, and measurements were performed at room temperature. The refractive indices of the particles and dispersant were set to 1.52 and 1.33, respectively.

### 2.6. Determination of Amylase Activity

Amylase activity of rice flour was determined using the DNS method, following previously reported procedures with slight modifications [16,17]. Rice flour (0.5 g) was suspended in 10 mL phosphate buffer (pH 6.0) and extracted at 25 °C for 20 min. The mixture was centrifuged at 4000× *g* for 10 min, and the supernatant was collected as the crude enzyme extract.

For total amylase activity, the reaction mixture consisted of 1 mL of crude enzyme extract, 1 mL of 1% (*w*/*v*) soluble starch solution, and 1 mL phosphate buffer (pH 6.0). The reaction was carried out at 40 °C for 10 min and terminated by adding 1 mL DNS reagent. The mixture was boiled for 10 min, rapidly cooled in an ice-water bath, diluted with distilled water to a final volume of 20 mL, and the absorbance was measured at 540 nm.

For α-amylase activity, β-amylase in the crude enzyme extract was first inactivated by heating at 70 °C for 15 min, followed by equilibration at 40 °C for 10 min. Subsequently, 1 mL of the treated enzyme extract was mixed with 1 mL of 1% (*w*/*v*) soluble starch solution and incubated at 40 °C for 5 min. The reaction was terminated by adding 2 mL of 0.4 M NaOH and 2 mL DNS reagent, followed by boiling for 10 min. After cooling, the reaction mixture was diluted to 20 mL, and absorbance was measured at 540 nm. A glucose standard curve was used for quantification. Amylase activity was expressed as units (U/g), defined as the amount of enzyme releasing 1 mg glucose per gram of dry sample per minute at 40 °C.

### 2.7. Determination of Pasting Properties

The pasting properties of rice flour were determined using a Rapid Visco Analyzer (RVA, Newport Scientific, Warriewood, NSW, Australia). Rice flour samples (3 g) were mixed with 25 g of deionized water in RVA aluminum cans [18]. The test program was set as follows: samples were equilibrated at 50 °C for 1 min, and the paddle was rotated at 960 rpm for 10 s to ensure complete dispersion, after which the speed was reduced to 160 rpm for the remainder of the test. The samples were heated from 50 to 95 °C at 5 °C/min, held at 95 °C, and then cooled to 50 °C and maintained for 2 min. The following RVA parameters were recorded: peak viscosity (PV), trough viscosity (TV), breakdown viscosity (BV), final viscosity (FV), setback viscosity (SV), and pasting temperature (PT).

### 2.8. Textural Profile Analysis (TPA)

The textural properties of cooked rice cooled to room temperature were measured using a texture analyzer (TA-XTplus, Stable Micro Systems, Surrey, UK) operated in two-cycle compression mode, following a previously described method [19]. For each measurement, 8 g of cooked rice was weighed and placed into a circular mold to form a compact rice cake, ensuring uniform sample thickness and complete coverage of the probe contact area. Samples were compressed using a 36 mm cylindrical probe (P/36R) at a pre-test, test, and post-test speed of 1 mm/s to a target deformation of 50% strain, with a 1 s interval between the two compression cycles. Measurements were performed using a 5 kg load cell, and at least six replicates were conducted for each sample.

### 2.9. X-Ray Diffraction (XRD)

The relative crystallinity (RC) of cooked rice samples harvested at different times was analyzed using a D8 Advance XRD diffractometer (Bruker-AXS, Billerica, MA, USA). Diffraction patterns were recorded over a 2θ range of 8–40° at a scanning rate of 12°/min. The RC was calculated based on the ratio of the integrated area of crystalline peaks to the total diffraction area, according to Equation (1):

(1) $$RC=\frac{\sum_{i=1}^{n}AUCi}{AUCt}$$

where AUC_i_ represents the area under the *i*-th crystalline peak, while AUC_t_ represents the total area of the diffraction pattern.

### 2.10. In Vitro Starch Digestibility

The in vitro small intestinal digestion of cooked rice flour was performed following a previously reported method [20]. Approximately 50 mg of the cooked rice sample was mixed with 2 mL of distilled water and incubated at 37 °C with stirring at 300 rpm. Subsequently, 8 mL of enzyme solution (preheated to 37 °C) containing 0.33 mg of pancreatin and 16.7 μL of amyloglucosidase in sodium acetate buffer (0.2 M, pH 6.0) was added. At predetermined time points (0, 5, 10, 15, 20, 30, 45, 60, 90, and 120 min), 100 μL aliquots were withdrawn and immediately mixed with 900 μL of absolute ethanol to terminate enzyme activity. The mixture was centrifuged at 3000 rpm for 10 min, and 50 μL of the supernatant was collected and mixed with 1.5 mL of GOPOD reagent. After incubation at 50 °C for 20 min, absorbance was measured at 510 nm. The glucose concentration was determined using a calibration curve prepared with standard glucose solutions. The amount of released glucose was converted to starch digested using a factor of 0.9 to account for the stoichiometric difference between glucose and anhydroglucose units in starch. Starch digestibility (%) was calculated based on the proportion of digested starch relative to the initial starch content of the sample.

Furthermore, starch digestion kinetics were analyzed following the method reported in previous studies [21]. The number of starch digestion stages was first assessed using the logarithm of slopes (LOS) plot based on first-order kinetics. To reduce noise introduced by numerical differentiation, non-linear least squares (NLLS) fitting was applied, with an overall fitting criterion of R^2^ ≥ 0.96, to validate the LOS results. The combined reaction kinetics (CRK) model was then used to quantify enzyme binding and catalytic processes during starch digestion [22], as described by Equations (2)–(4).

For starch substrate:

(2) $$C_{SS}(t)=(C_{DP}(\infty ))\times (e^{-k_{b}t})$$

For the enzyme–starch complex:

(3) $$C_{ES}\left(t\right)=\frac{k_{b}\times C_{DP}\left(\infty \right)}{k_{cat}-k_{b}}\times \left(e^{-k_{b}t}-e^{-k_{cat}t}\right)$$

For the starch digested product:

(4) $$C_{DP}\left(t\right)=C_{DP}\left(\infty \right)\times \left(1-\frac{k_{cat}\times e^{-k_{b}t}-k_{b}\times e^{-k_{cat}t}}{k_{cat}-k_{b}}\right)$$

where C_SS_(t), C_ES_(t), and C_DP_(t) represent the amounts of starch substrate, enzyme–starch complex, and digested product at time t, respectively. K_b_ and K_cat_ denote the enzyme binding and catalysis rate coefficients, and C_DP_(∞) is obtained by NLLS fitting of the digestion curves using the CRK model and is interpreted as an apparent asymptotic parameter describing the overall digestion trend.

### 2.11. Statistical Analysis

All experiments were performed at least in triplicate, and results are expressed as mean ± standard deviation. Statistical analysis was carried out using IBM SPSS Statistics 25 (IBM, Armonk, NY, USA). Differences among samples were evaluated by one-way analysis of variance (ANOVA), and mean comparisons were performed using Tukey’s test. A significance level of *p* < 0.05 was applied. Figures were prepared using Origin 2021 software (OriginLab, Northampton, MA, USA).

## 3. Results and Discussion

### 3.1. Protein Content

Protein is a fundamental chemical component that determines rice grain structure and subsequent physicochemical behavior during cooking and digestion [6,23]. As shown in Table 1, harvest time significantly affected the protein content of WYD4 rice (*p* < 0.05). The protein content increased gradually from 8.18% at week 1 to a maximum of 8.81% at week 4, followed by a significant decline at weeks 6 and 7. The decrease in protein content with delayed harvest can be explained by the dynamic changes during grain maturation. In the early stages, storage protein accumulation occurs first, whereas the later grain-filling phase is dominated by rapid starch biosynthesis and deposition. As starch progressively accumulates within the endosperm, its proportion increases, leading to a relative dilution of protein content in the grain. Protein plays an important role in determining rice cooking behavior due to its strong water-binding capacity [24]. Higher protein content generally requires more water and longer cooking time, and excessive protein is associated with increased surface hardness of cooked rice [25]. Previous studies have reported that high-protein rice exhibits significantly higher hardness than low-protein rice, which may negatively affect eating quality [26]. Therefore, the observed lower protein content at early and late harvest times may partially contribute to the improvement of textural softness.

### 3.2. Amylose Content and Starch Chain Length Distributions (CLDs)

To further elucidate the molecular structural changes in starch induced by different harvest times, the CLDs of debranched starch were analyzed (Table 1). Starch chains with degree of polymerization (DP) ≤ 100 were assigned to amylopectin, whereas chains with DP > 100 were classified as amylose. Amylose content fluctuated with harvest time but remained within the intermediate range (18–24%) throughout all stages. It decreased from 20.38% at week 1 to the lowest value of 18.13% at week 5, followed by a significant increase at weeks 6 and 7, with the highest amylose content observed at week 6 (23.57%). Rice with intermediate amylose content is generally preferred by consumers due to its balanced softness and stickiness [27]. Amylose is also a key determinant of starch retrogradation and digestibility. Higher amylose content is positively correlated with cooked rice hardness and reduced stickiness [28]. Moreover, starch chains with DP between 100 and 500 (defined as short- to intermediate-length amylose chains in this study) showed an overall decreasing trend with delayed harvesting, with the highest value at week 1 (3.50%) and the lowest value at week 6 (1.93%). In contrast, longer amylose chains (DP 500–5000 and DP 5000–20,000) did not exhibit a clear monotonic trend across harvest periods, suggesting that harvest time primarily regulates the proportion of short- and intermediate-length amylose chains rather than extremely long amylose chains.

The proportion of amylopectin short chains (DP 6–12) showed no significant difference among all harvest times (*p* > 0.05), ranging from 18.23% to 19.41%. In contrast, the proportion of amylopectin medium chains (DP 13–24) was significantly affected by harvest time (*p* < 0.05). The highest value was observed at week 2 (27.31%), which was significantly higher than that at week 1 (24.31%) and week 7 (25.66%). The proportion of amylopectin long chains (DP 36–100) accounted for more than 30% of total starch in all samples, confirming their dominant role in the starch molecular architecture of WYD4 rice. Notably, this fraction exhibited an overall increasing trend with delayed harvesting, rising from 30.33% at week 1 to the highest value of 32.72% at week 6. Long amylopectin chains are known to promote stronger intermolecular interactions and facilitate double-helix formation, which contributes to increased rigidity of the starch network [29]. Therefore, the accumulation of long-chain amylopectin at later harvest stages, together with the elevated amylose content at weeks 6–7, may partially explain the enhanced swelling capacity of starch granules and the secondary rise in retrogradation tendency observed in the pasting properties analysis. It should be noted that these changes were observed in a single japonica rice variety (WYD4), and the magnitude and specific patterns of CLD variation may depend on genetic background. Collectively, these results demonstrate that delayed harvesting promotes the accumulation of long amylopectin chains, leading to a more rigid starch molecular architecture.

### 3.3. Particle Size Distribution

**Figure 1:**
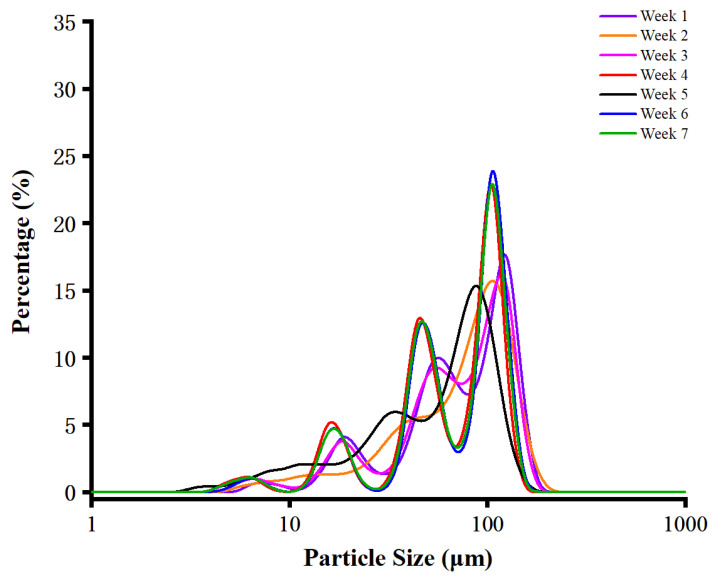
Particle size distribution of WYD4 rice flour at different harvest times.

Particle size distribution reflects the physical manifestation of internal starch-protein organization and plays an important role in determining flour functionality [30,31,32]. As shown in Figure 1 and Table 2, harvest time significantly affected all particle size parameters of WYD4 rice (*p* < 0.05). With delayed harvesting, the average particle size exhibited an overall decreasing trend, declining from 74.37 μm at week 1 to 66.85 μm at week 7. This reduction in average particle size with grain maturation is essentially attributed to the dynamic changes in endosperm hardness during the post-heading stage. Rice endosperm hardness is defined by the resistance of the starch-protein matrix to mechanical deformation during milling, and it is closely associated with the continuity of the protein matrix and the adhesion between starch granules and protein components [33]. During late grain maturation (delayed harvesting), continuous starch accumulation and granule formation in the endosperm progressively disrupt the original continuous network structure of the protein matrix; this structural dissociation weakens the adhesion between starch granules and the protein matrix, leading to a decrease in endosperm hardness. A softer endosperm is more easily disintegrated into small particles during the milling process, thus resulting in a smaller average particle size of rice flour [34]. In addition, the gradual decrease in protein content at late harvest time (Table 1) further reduces the physical cross-linking of the starch-protein matrix, which also contributes to the reduction in endosperm hardness and the subsequent decrease in particle size. Moreover, particle size is closely related to enzymatic accessibility, as smaller particles provide a larger specific surface area for enzyme binding and diffusion [32,35]. Therefore, the progressive reduction in particle size with grain maturation is expected to enhance amylase-starch interactions and subsequently accelerate starch digestion, which is consistent with the digestion results reported later.

The D10 values of rice harvested at weeks 1 and 2 were significantly higher, suggesting that the finer particles were generally larger at early harvest times. A similar trend was observed for D90, indicating that both the lower and upper ends of the particle size distribution shifted toward larger particle sizes during early harvesting. These results suggest that harvest time markedly alters rice flour particle morphology, with a gradual decline in both fine and coarse aggregated particles as grain maturation progresses. The reduction in average particle size and coarse-particle proportion at later harvest time is expected to enhance enzymatic accessibility by increasing the effective surface area for enzyme binding [32]. Consequently, such structural changes may contribute to modified pasting behavior and accelerated starch digestion kinetics observed at advanced harvest time [32,36].

### 3.4. The Effect of Harvest Time on the Amylase Activity

**Figure 2:**
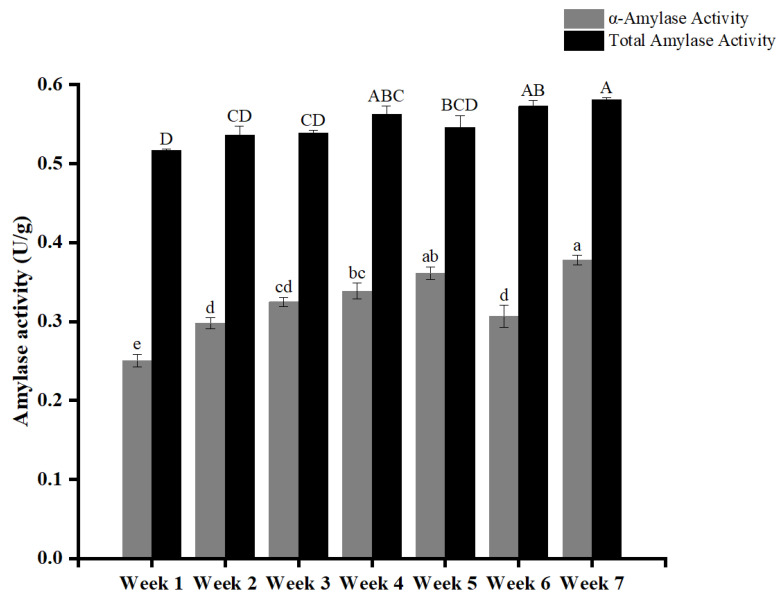
Effects of harvest time on α-amylase and total amylase activities in WYD4 rice. Different lowercase and uppercase letters indicate significant differences in α-amylase and total amylase activities, respectively (*p* < 0.05).

The activities of α-amylase and total amylase in WYD4 rice harvested at different time points are shown in Figure 2. Harvest time significantly affected all enzymatic activities (*p* < 0.05), indicating that grain maturity strongly regulates endogenous starch-degrading enzymes. Both total amylase and α-amylase exhibited the same increasing trend with delayed harvesting. Total amylase activity increased gradually from 0.516 U/g at week 1 to 0.581 U/g at week 7, while α-amylase activity rose from 0.251 U/g to 0.378 U/g over the same period. This parallel trend indicates that α-amylase is the major contributor to the increase in total amylase activity during late harvest time. α-Amylase plays a central role in starch depolymerization by randomly cleaving α-1,4-glycosidic bonds, producing dextrins and oligosaccharides [37,38]. During seed germination, this enzyme mobilizes stored starch reserves to provide metabolic energy [39]. After harvest, α-amylase activity is closely associated with the physiological status of the grain, which in this study reflects its intrinsic developmental maturity and biochemical state at different harvest times. A previous study [40] reported that α-amylase gradually reshapes the multi-scale structure of rice starch through a cumulative degradation effect during storage, leading to enhanced gelation behavior and accelerated digestibility. Consistent with these findings, the progressive increase in α-amylase and total amylase activities observed in the present study suggests that delayed harvesting promotes continuous enzymatic modification of starch. From a quality perspective, elevated amylase activity may be unfavorable for storage stability, as excessive starch hydrolysis can weaken granule integrity and accelerate quality deterioration [40].

### 3.5. Pasting Properties

RVA pasting properties reflect the combined effects of starch molecular structure, protein content, and granule organization on gelatinization and retrogradation behavior during heating and cooling [41]. As shown in Table 3, harvest time significantly affected all RVA parameters, indicating that grain maturity alters starch functional behavior. Although individual RVA parameters did not exhibit a simple monotonic trend, their variations showed clear associations with changes in starch molecular structure and composition.

PV, which reflects the swelling capacity of starch granules, was relatively stable during weeks 1–5 but increased significantly at weeks 6–7. This increase coincided with the higher proportion of long amylopectin chains (DP > 36) and the increase in amylose content observed at later harvest time. Long amylopectin chains are known to enhance granule swelling due to stronger inter-chain interactions, while increased amylose may contribute to higher paste viscosity through network formation [42]. Therefore, the elevated PV at later harvest time can be mechanistically attributed to molecular restructuring of starch chains rather than random variation.

BV, an indicator of paste stability under shear and heat, remained relatively low during weeks 1–3, corresponding to the moderate protein content and shorter amylopectin chain distribution observed in early-harvested rice. Proteins can restrict granule swelling and enhance paste stability by forming a physical barrier around starch granules [42]. The relatively low BV values at early harvest times therefore indicate greater thermal stability, which is desirable for processing applications requiring prolonged heating.

SV, which reflects starch retrogradation tendency, showed higher values at intermediate harvest time (weeks 3–4). This behavior is consistent with the balanced amylose content observed during this period. Amylose is the primary contributor to retrogradation due to its strong tendency to reassociate during cooling [43]. The increase in amylose content at later harvest time (weeks 6–7) further explains the secondary rise in SV, confirming that amylose chain reorganization is the dominant driver of retrogradation behavior.

Importantly, the absence of a simple monotonic trend in RVA parameters reflects the dynamic interaction between protein content and starch molecular architecture during grain maturation. Early harvest times are characterized by higher protein content and shorter amylopectin chains, which jointly contribute to improved paste stability and reduced retrogradation. In contrast, delayed harvesting promotes accumulation of long amylopectin chains, leading to enhanced swelling capacity but increased retrogradation tendency. Overall, these results demonstrate that harvest time regulates rice pasting behavior primarily through modulating starch molecular structure and protein-starch interactions, rather than through simple maturity-driven trends. From a functional perspective, rice harvested during weeks 1–3 exhibited more stable pasting characteristics, supporting their suitability for high-quality processing and cooking applications.

### 3.6. Texture Profile Analysis (TPA) of Cooked Rice

Texture profile analysis (TPA) directly reflects the eating quality of cooked rice, among which hardness and stickiness are the most critical determinants of consumer acceptance [44]. As shown in Table 4, harvest time significantly affected most textural parameters of WYD4 rice, indicating that grain maturity plays an important role in shaping cooked rice texture.

Hardness showed significant variation among harvest times. The highest hardness was observed at week 1, whereas the lowest value occurred at week 7. This indicates that early-harvested rice produced a firmer cooked texture, while delayed harvesting resulted in softer rice. This difference can be mechanistically explained by changes in protein content and starch structure. As shown in Table 1, protein content decreased after week 4, and lower protein levels reduce the physical restriction on starch granule swelling, leading to softer texture [45,46]. In addition, rice harvested at later stages exhibited higher PV, which is associated with reduced hardness. Previous studies have reported a significant negative correlation between PV and cooked rice hardness [44], supporting the present observations.

Adhesiveness (stickiness) also varied significantly with harvest time. The highest adhesiveness was observed at week 2, whereas the lowest value occurred at week 4. However, no consistent relationship was observed between adhesiveness and amylose content. This finding agrees with previous studies indicating that rice stickiness is regulated by multiple factors rather than amylose content alone [44]. Protein composition and fine starch molecular structure are also important contributors. Previous studies have reported that the ratio of prolamin to glutelin is positively correlated with rice stickiness, suggesting that protein fractions may play a dominant role [46]. In addition, variations in amylopectin chain length distribution may also influence surface adhesion behavior.

Other textural parameters, including cohesiveness, gumminess, chewiness, and resilience, also showed significant differences among harvest times (*p* < 0.05). Early harvested rice (weeks 1–3) generally exhibited higher gumminess and chewiness, consistent with its higher protein content and more compact starch structure, whereas rice harvested at later stages showed lower values, reflecting a softer and less dense texture. Overall, these results indicate that harvest time modulates cooked rice texture mainly through coordinated changes in protein content, starch molecular architecture, and pasting behavior. Rather than following a simple monotonic trend, textural properties exhibited stage-dependent variations, highlighting the complex interactions among grain composition, structure, and cooking behavior.

### 3.7. Harvest Time Affects the Crystallinity of Cooked WYD4 Rice

**Figure 3:**
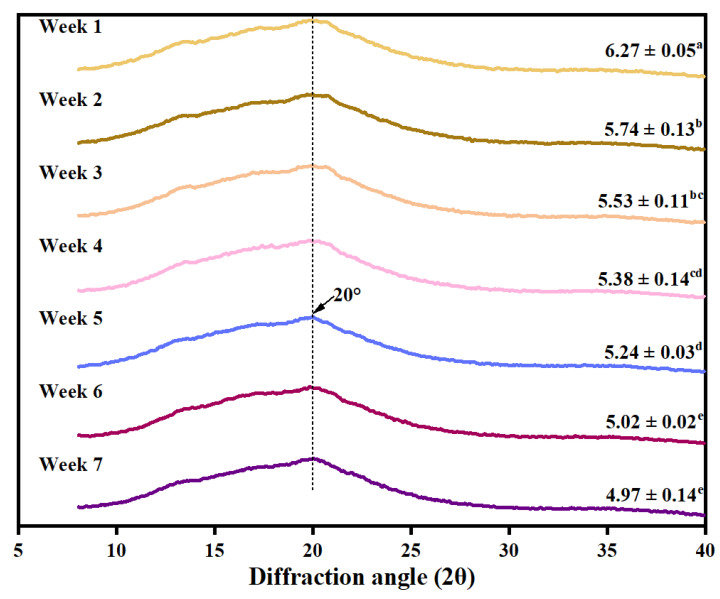
X-ray diffraction (XRD) patterns and relative crystallinity of cooked WYD4 rice harvested at different times. Different letters (a–e) indicate significant differences among treatments (*p* < 0.05).

The X-ray diffraction (XRD) patterns of cooked rice harvested at different stages are shown in Figure 3. All samples exhibited a typical V-type crystalline structure, which is characteristic of amylose-lipid complexes formed during the cooking process [47]. Relative crystallinity decreased significantly and continuously with delayed harvesting, declining from 6.27% at week 1 to 4.97% at week 7. This result suggests that harvest time strongly influences the ordered arrangement of starch molecules in cooked rice. The delayed harvesting altered starch chain length distribution, characterized by an increased proportion of long-chain amylopectin (Table 1). These structural features interfere with the re-association of starch chains into tightly packed double helices after gelatinization, thereby suppressing the reconstruction of crystalline domains and resulting in lower crystallinity. Meanwhile, the elevated enzyme activity at later harvest time may hydrolyze α-1,4-glycosidic bonds and gradually disrupt crystalline lamellae, further weakening ordered starch regions [38]. Moreover, smaller particle size increases specific surface area and facilitates enzyme penetration, which enhances enzymatic accessibility to starch chains and accelerates structural breakdown during cooking. Furthermore, the decline in protein content observed after week 4 may reduce the physical constraints surrounding starch granules, allowing greater molecular mobility and structural relaxation. Together, these factors may act synergistically to drive the continuous decrease in starch crystallinity with delayed harvesting.

### 3.8. Starch Digestibility of WYD4 Rice

**Figure 4:**
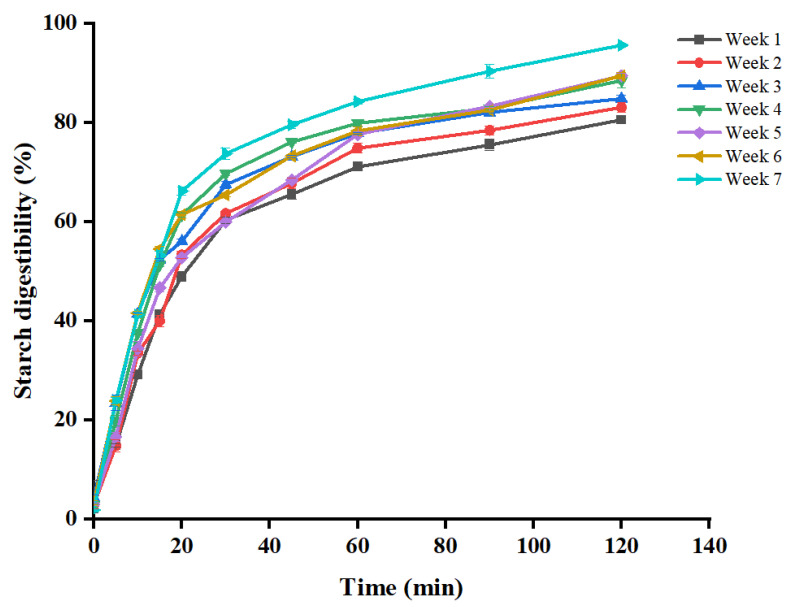
In vitro starch digestograms of cooked WYD4 rice harvested at different times.

**Figure 5:**
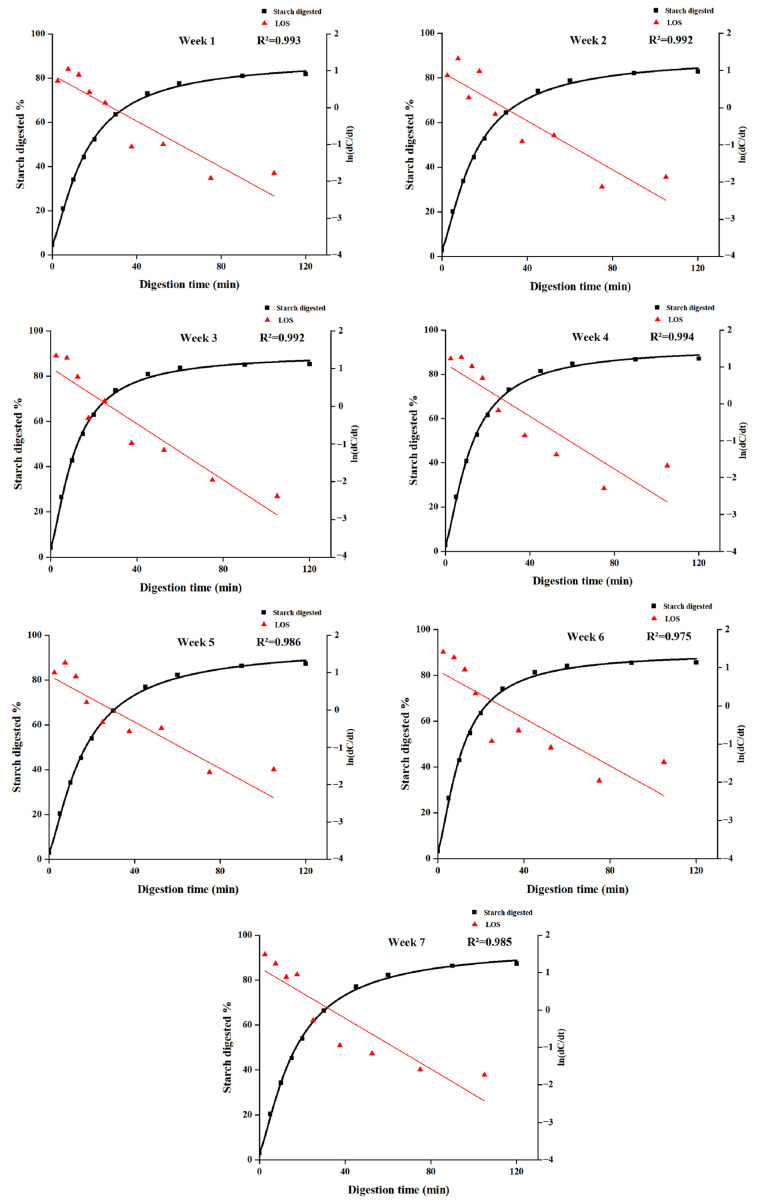
Logarithm of slopes (LOS) plots and simple first-order kinetic fittings of starch digestion profiles for WYD4 rice harvested at different times.

**Figure 6:**
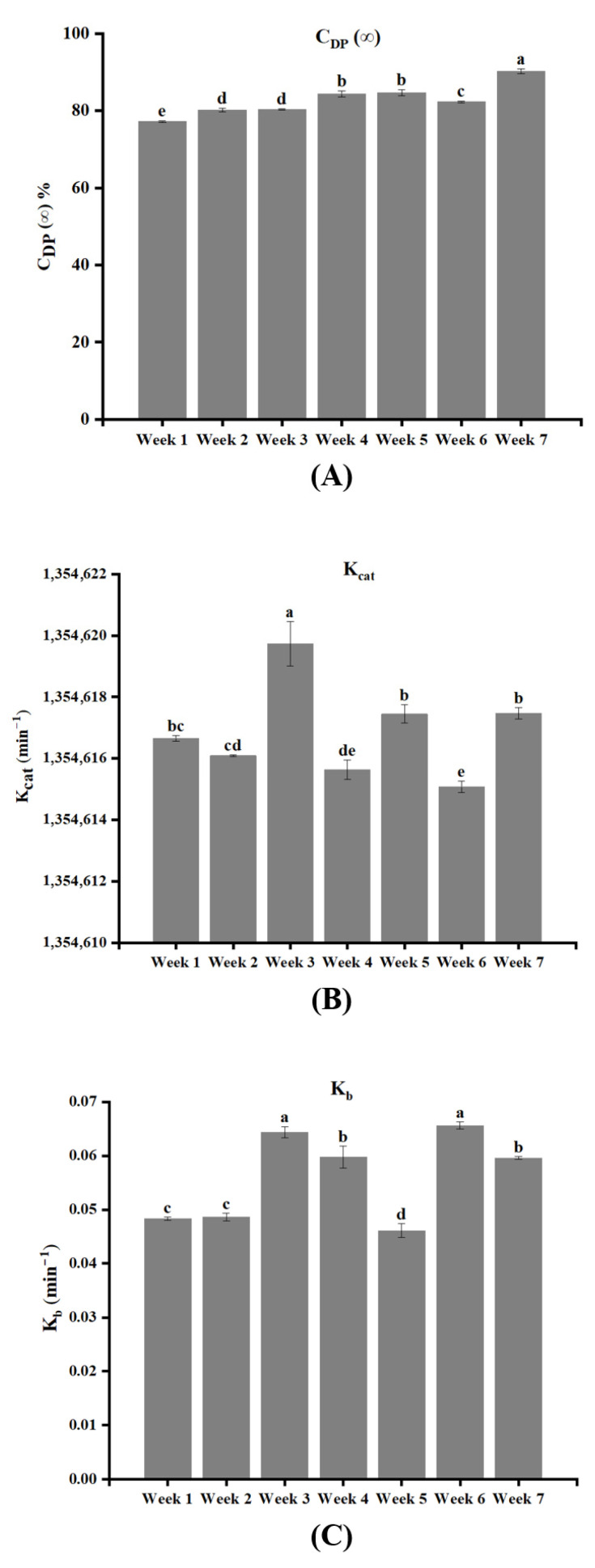
Effects of harvest time on apparent asymptotic digestion extent (C_DP_(∞), (**A**)), catalytic rate constant (K_cat_, (**B**)), and enzyme binding rate constant (K_b_, (**C**)) of WYD4 rice obtained from combined reaction kinetics (CRK) modeling. Different letters (a–e) indicate significant differences among treatments (*p* < 0.05).

Significant differences in starch digestibility were observed among rice samples harvested at different stages (Figure 4). The 120 min digestion extent increased progressively from 80.53% at week 1 to 95.60% at week 7, indicating that delayed harvesting markedly enhanced starch digestibility. LOS plots showed good linearity (R^2^ ≥ 0.97), indicating that starch digestion followed a single kinetic stage across all harvest times (Figure 5). Accordingly, the CRK model was applied to quantify enzyme binding and catalytic parameters (Figure 6). The enzyme binding rate constant (K_b_) was consistently lower than the catalytic rate constant (K_cat_), confirming that enzyme binding is the rate-limiting step during rice starch digestion. With delayed harvesting, K_b_ exhibited an overall increasing trend, indicating enhanced enzyme-substrate affinity. Meanwhile, the fitted $C_{DP}(\infty )$ values increased with delayed harvesting, indicating enhanced overall digestibility of rice starch. This trend is consistent with the increase in endogenous amylase activity, the decrease in starch crystallinity, and the reduction in particle size observed at later harvest time, all of which facilitate enzyme accessibility and accelerate starch hydrolysis. Changes in starch CLDs also contributed to the digestion behavior. Delayed harvesting altered the proportions of amylopectin and amylose chains, which can modify molecular packing and short- and long-range ordered structures after gelatinization [48]. Previous studies have shown that variations in starch CLDs regulate digestibility mainly by affecting crystalline organization and enzyme accessibility, rather than through a simple linear relationship with chain length [49,50]. In the present study, the harvest-dependent structural rearrangements in CLDs likely weakened molecular order and promoted enzymatic hydrolysis, consistent with the observed increase in digestibility. It should be noted that $C_{DP}(\infty )$ represents an apparent asymptotic parameter derived from global fitting of the digestion curves and may slightly deviate from individual experimental values at specific time points, particularly when a clear plateau is not fully reached within the experimental duration.

Starch digestibility is closely associated with the glycemic index (GI), with faster digestion generally leading to a higher postprandial glucose response [51]. Therefore, the increased digestibility observed at later harvest time suggests a potential increase in predicted GI, which may be unfavorable for glycemic control [51,52]. High GI diets have been linked to an elevated risk of type 2 diabetes, particularly in populations with high rice consumption [53]. Accordingly, rice harvested at earlier stages may contribute to a more moderate glycemic response.

## 4. Conclusions

In conclusion, this study systematically investigated the effects of harvest time on the physicochemical properties, structural characteristics, and starch digestion behavior of the aromatic japonica rice cultivar WYD4. Delayed harvesting significantly altered rice composition and structure, as reflected by changes in protein and amylose contents, starch CLDs, particle size, amylase activity, and starch crystallinity. These coordinated structural and enzymatic modifications collectively enhanced starch digestibility. Although delayed harvesting reduces hardness and results in a softer texture, this does not necessarily correspond to improved overall eating quality. In contrast, earlier harvest times are associated with lower amylase activity, slower starch digestion, and more stable structural and physicochemical properties. These characteristics contribute to better quality stability and potential nutritional advantages, such as a lower digestion rate. Therefore, considering both eating quality and nutritional aspects, earlier harvest times (approximately weeks 1–3) are suggested as more favorable overall. These findings provide a practical basis for optimizing harvest strategies in WYD4 rice production.

## Figures and Tables

**Table 1:** Basic components and starch chain length distributions (CLDs) of WYD4 rice harvested at different times.

Harvest Time	Protein Content (%)	Amylose Content (%)	DP (6–12)	DP (13–24)	DP (25–36)	DP (36–100)	DP (100–500)	DP (500–5000)	DP (5000–20,000)
Week 1	8.18 ± 0.05 ^bc^	20.38 ± 3.27 ^ab^	19.41 ± 1.76 ^a^	24.31 ± 0.41 ^c^	9.48 ± 0.27 ^ab^	30.33 ± 0.58 ^a^	3.50 ± 2.01 ^a^	7.32 ± 0.11 ^a^	5.65 ± 0.79 ^a^
Week 2	8.31 ± 0.02 ^abc^	19.84 ± 0.50 ^ab^	18.23 ± 1.39 ^a^	27.31 ± 1.18 ^a^	9.85 ± 0.16 ^b^	30.46 ± 0.51 ^ab^	2.75 ± 0.94 ^a^	7.58 ± 0.12 ^b^	3.82 ± 0.19 ^a^
Week 3	8.50 ± 0.01 ^ab^	19.60 ± 2.32 ^ab^	19.00 ± 0.53 ^a^	26.68 ± 0.48 ^ab^	8.84 ± 0.26 ^b^	30.69 ± 0.56 ^bc^	2.93 ± 0.42 ^a^	7.31 ± 0.21 ^ab^	4.55 ± 0.55 ^a^
Week 4	8.81 ± 0.50 ^a^	21.89 ± 2.22 ^ab^	18.59 ± 0.36 ^a^	26.94 ± 0.95 ^ab^	9.71 ± 0.18 ^ab^	30.43 ± 0.50 ^abc^	2.84 ± 1.17 ^a^	7.61 ± 0.18 ^b^	3.88 ± 0.63 ^a^
Week 5	8.37 ± 0.03 ^ab^	18.13 ± 0.45 ^b^	18.62 ± 1.30 ^a^	26.24 ± 0.25 ^ab^	9.43 ± 0.25 ^ab^	31.80 ± 0.49 ^ab^	2.05 ± 0.56 ^a^	7.95 ± 0.77 ^c^	3.91 ± 0.60 ^a^
Week 6	7.74 ± 0.04 ^c^	23.57 ± 1.93 ^a^	19.12 ± 0.23 ^a^	25.77 ± 1.56 ^bc^	9.47 ± 0.46 ^a^	32.72 ± 0.76 ^bc^	1.93 ± 0.92 ^a^	7.57 ± 0.06 ^a^	3.42 ± 0.40 ^a^
Week 7	7.95 ± 0.04 ^bc^	22.55 ± 0.50 ^ab^	18.91 ± 0.53 ^a^	25.66 ± 0.30 ^b^	8.94 ± 0.28 ^ab^	32.51 ± 0.47 ^c^	2.61 ± 0.46 ^a^	7.63 ± 0.14 ^ab^	3.74 ± 0.05 ^a^

Note: Values are expressed as mean ± standard deviation (SD). Different superscript letters within the same column indicate significant differences (*p* < 0.05).

**Table 2:** Particle size distribution parameters (mean particle size, D10, and D90) of WYD4 rice flour harvested at different times.

Harvest Time	Mean Particle Size (μm)	D10 (μm)	D90 (μm)
Week 1	74.37 ± 1.07 ^a^	18.94 ± 0.59 ^b^	122.53 ± 1.31 ^a^
Week 2	73.49 ± 0.53 ^a^	22.99 ± 0.49 ^a^	120.53 ± 0.62 ^b^
Week 3	70.49 ± 0.12 ^b^	17.33 ± 0.05 ^c^	118.13 ± 0.42 ^c^
Week 4	64.41 ± 0.07 ^e^	15.33 ± 0.09 ^e^	103.23 ± 0.05 ^e^
Week 5	56.82 ± 0.78 ^f^	12.53 ± 0.55 ^f^	97.50 ± 0.78 ^f^
Week 6	68.85 ± 0.66 ^c^	16.24 ± 0.30 ^d^	108.57 ± 0.17 ^d^
Week 7	66.85 ± 0.36 ^d^	15.90 ± 0.23 ^de^	106.87 ± 0.62 ^d^

Note: Values are expressed as mean ± standard deviation (SD). Different superscript letters within the same column indicate significant differences (*p* < 0.05). D10 represents the particle size below which 10% of the particles fall (fine particles), and D90 represents the particle size below which 90% of the particles fall (coarse particles).

**Table 3:** Pasting properties of WYD4 rice harvested at different times.

Harvest Time	PV (cP)	TV (cP)	BV (cP)	FV (cP)	SV (cP)	PT (°C)
Week 1	2750.67 ± 5.44 ^cd^	1934.33 ± 36.97 ^de^	805.00 ± 37.5 ^ab^	2994.33 ± 27.92 ^bc^	1056.33 ± 11.03 ^bc^	72.38 ± 0.45 ^ab^
Week 2	2808.67 ± 20.73 ^c^	2057.00 ± 7.48 ^c^	756.00 ± 12.68 ^b^	3067.00 ± 6.16 ^b^	1008.00 ± 14.35 ^e^	72.70 ± 0.00 ^a^
Week 3	2682.00 ± 39.05 ^d^	1905.50 ± 9.42 ^de^	768.00 ± 47.08 ^b^	2979.50 ± 3.86 ^c^	1074.00 ± 3.40 ^ab^	72.63 ± 0.02 ^a^
Week 4	2683.33 ± 47.15 ^d^	1881.50 ± 25.96 ^e^	819.00 ± 27.78 ^ab^	2982.00 ± 29.17 ^bc^	1100.50 ± 2.05 ^a^	71.88 ± 0.02 ^b^
Week 5	2693.00 ± 48.36 ^d^	1951.00 ± 20.14 ^d^	739.00 ± 34.29 ^b^	2992.50 ± 23.33 ^bc^	1041.50 ± 5.31 ^cd^	72.00 ± 0.12 ^ab^
Week 6	3052.33 ± 2.05 ^a^	2202.50 ± 48.87 ^a^	848.50 ± 50.47 ^a^	3223.50 ± 63.51 ^a^	1021.00 ± 17.91 ^de^	72.23 ± 0.38 ^ab^
Week 7	2931.67 ± 52.85 ^b^	2143.50 ± 21.51 ^b^	771.00 ± 29.94 ^b^	3219.00 ± 32.29 ^a^	1075.50 ± 11.15 ^b^	72.25 ± 0.42 ^ab^

Note: Values are expressed as mean ± standard deviation (SD). Different superscript letters within the same column indicate significant differences (*p* < 0.05). PV, peak viscosity; TV, trough viscosity; BV, breakdown viscosity; FV, final viscosity; SV, setback viscosity; PT, pasting temperature.

**Table 4:** Effects of harvest time on textural properties of cooked WYD4 rice.

Harvest Time	Hardness (g)	Adhesiveness (g·s)	Springiness	Cohesiveness	Gumminess	Chewiness
Week 1	1772.87 ± 71.68 ^a^	−414.86 ± 16.79 ^bc^	0.91 ± 0.02 ^a^	0.46 ± 0.01 ^a^	816.26 ± 33.07 ^a^	743.72 ± 23.75 ^a^
Week 2	1694.23 ± 47.45 ^ab^	−416.11 ± 12.39 ^bc^	0.88 ± 0.07 ^a^	0.44 ± 0.03 ^ab^	712.52 ± 49.01 ^b^	693.33 ± 24.46 ^ab^
Week 3	1596.41 ± 62.42 ^bc^	−323.05 ± 24.02 ^a^	0.88 ± 0.04 ^a^	0.42 ± 0.01 ^ab^	673.07 ± 12.06 ^bc^	595.19 ± 35.04 ^c^
Week 4	1532.10 ± 53.82 ^c^	−316.39 ± 19.30 ^a^	0.91 ± 0.01 ^a^	0.42 ± 0.02 ^ab^	629.44 ± 5.94 ^bc^	548.16 ± 33.01 ^c^
Week 5	1674.45 ± 55.56 ^ab^	−322.47 ± 40.57 ^a^	0.92 ± 0.01 ^a^	0.40 ± 0.02 ^ab^	672.82 ± 19.54 ^bc^	616.02 ± 18.99 ^bc^
Week 6	1605.65 ± 48.41 ^bc^	−344.78 ± 27.09 ^ab^	0.84 ± 0.04 ^a^	0.43 ± 0.02 ^ab^	696.11 ± 31.09 ^bc^	584.02 ± 39.18 ^c^
Week 7	1527.77 ± 41.79 ^c^	−374.72 ± 48.25 ^c^	0.85 ± 0.00 ^a^	0.39 ± 0.01 ^b^	614.86 ± 2.61 ^c^	553.10 ± 10.96 ^c^

Note: Values are expressed as mean ± standard deviation (SD). Different superscript letters within the same column indicate significant differences (*p* < 0.05).

## Data Availability

The original contributions presented in this study are included in the article. Further inquiries can be directed to the corresponding author.
